# Anti-angiogenic VEGFAxxxb transcripts are not expressed in the medio-basal hypothalamus of the seasonal sheep

**DOI:** 10.1371/journal.pone.0197123

**Published:** 2018-05-10

**Authors:** Didier Lomet, Benoît Piégu, Shona H. Wood, Hugues Dardente

**Affiliations:** 1 PRC, INRA, CNRS, IFCE, Université de Tours, Nouzilly, France; 2 Dept of Arctic and Marine Biology UiT–The Arctic University of Norway, Tromsø, Norway; University College London School of Pharmacy, UNITED KINGDOM

## Abstract

This study investigated *Vegfa* expression in the *pars tuberalis* (PT) of the pituitary and medio-basal hypothalamus (MBH) of sheep, across seasons and reproductive states. It has recently been proposed that season impacts alternative splicing of *Vegfa* mRNA in the PT, which shifts the balance between angiogenic VEGFAxxx and anti-angiogenic VEGFAxxxb isoforms (with xxx the number of amino acids of the mature VEGFA proteins) to modulate seasonal breeding. Here, we used various RT-PCR methodologies and analysis of RNAseq datasets to investigate seasonal variation in expression and splicing of the ovine *Vegfa* gene. Collectively, we identify 5 different transcripts for *Vegfa* within the ewe PT/MBH, which correspond to splicing events previously described in mouse and human. All identified transcripts encode angiogenic VEGFAxxx isoforms, with no evidence for alternative splicing within exon 8. These findings led us to investigate in detail how “*Vegfaxxxb*-like” PCR products could be generated by RT-PCR and misidentified as endogenous transcripts, in sheep and human HEK293 cells. In conclusion, our findings do not support the existence of anti-angiogenic VEGFAxxxb isoforms in the ovine PT/MBH and shed new light on the interpretation of prior studies, which claimed to identify *Vegfaxxxb* isoforms by RT-PCR.

## Introduction

Our understanding of the molecular and neuroendocrine mechanisms that govern seasonal breeding has increased considerably over the last 20 years. In the current model, melatonin acts within the *pars tuberalis* (PT) of the pituitary to increase *Tshb* transcription under long days [1–4]. This PT-derived specific TSH (Thyrotropin-Stimulating Hormone) acts locally on specialized glial cells, known as tanycytes, to trigger the expression of thyroid hormone deiodinase type II (*Dio2*), which increases T3 levels within the medio-basal hypothalamus (MBH) during long days. This phylogenetically conserved mechanism underlies the critical role of hypothalamic thyroid hormone signaling in the control of seasonal cycles [5–7].

Beyond this TSH/DIO2/T3 signaling pathway, it has become clear that season impacts the expression of hundreds of genes in the PT/MBH region. The combination of transcriptomics and gene ontology analyses in sheep has pointed to several key pathways that include cellular plasticity, extracellular matrix remodeling, cell proliferation, epigenetics and angiogenesis [8,9,10]. In an unexpected twist, Castle-Miller *et al*. [11] recently proposed a role for PT-derived VEGFA in the seasonal regulation of pituitary angiogenesis in sheep [11–13]. Such changes are proposed to play a role in the control of seasonal breeding through the modulation of pituitary blood flow hence the access of hypothalamic releasing factors such as GnRH to the *pars distalis* (PD) of the pituitary. Specifically, the authors show that the *Vegfa* transcript undergoes seasonally-controlled alternative splicing of exon 8, which gives rise either to classical angiogenic *Vegfaxxx* isoforms (with xxx the number of amino acids of the mature VEGFA proteins) or to anti-angiogenic *Vegfaxxxb* isoforms [11]. This alternative splicing event of the exon 8 of *Vegfa* was initially described by Bates *et al*. for human tissues and would therefore likely be conserved across mammals [14]. Castle-Miller *et al*. proposed that the balance between both isoforms—angiogenic VEGFAxxx and anti-angiogenic VEGFAxxxb—is integral to the seasonal control of pituitary function [11].

Based on these findings, we investigated the expression profile and splicing of *Vegfa* transcripts, across seasons and reproductive states in sheep. Here, we report that both *Vegfa* and *VegfR2* display modest seasonal variation in their overall expression level in the PT/MBH, suggestive of enhanced VEGFA signaling during long days. We identify five different pro-angiogenic *Vegfaxxx* splice variants in the ovine MBH. The main splicing event corresponds to the skipping of exon 6, thereby leading to VEGFA164. We also identify transcripts in which both exons 6 and 7 are skipped (leading to VEGFA120) and transcripts in which cryptic splice sites within exon 6 are used. However, neither our extensive RT-PCR analysis nor our investigation of recent ovine RNAseq datasets [9,10] provided experimental support for the existence of endogenous anti-angiogenic *Vegfaxxxb* transcripts in the ovine PT/MBH, regardless of season or reproductive status. This suggests that such *Vegfaxxxb* transcripts are either extremely rare or that they do not exist at all, which makes it very unlikely that such anti-angiogenic isoforms play a significant role in seasonal physiology. Furthermore, using a step-by-step empirical approach to PCR primer design we provide evidence that detection of these isoforms in prior studies, including studies in human cell lines and tissues [14] might be artefactual, as previously suggested by Harris *et al*. [15].

Collectively, the results of our investigation into *Vegfa* splicing in the MBH of the sheep concur with prior findings in mouse and humans and suggest that mechanisms are phylogenetically conserved and lead only to the production of pro-angiogenic VEGFAxxx isoforms [12,13,16].

## Materials & methods

Apart from experiment 4 (see Experimental animals & procedures), the samples being analysed in the current study were collected in multiple cohorts, which have been used in previous experiments aimed at investigating the roles of photoperiod and thyroid hormone in seasonal breeding. For detailed accounts of these cohorts and experimental procedures, the reader is referred to a recently published study [10].

### Ethics statement

All experimental procedures were performed in accordance with international (directive 2010/63/UE) and national legislation (décret n° 2013–118) governing the ethical use of animals in research (authorization n° E37–175–2 and n°A38 801). All procedures used in this work were evaluated by a local ethics committee (Comité d’Ethique en Expérimentation Animale Val de Loire) and approved by the Ministry of Higher Education and Research (project n°00710.02). All surgeries were performed after sodium thiopental anesthesia (Nesdonal®, 1g/80kg), under constant isoflurane administration (Vetflurane®) and all efforts were made to minimize suffering. Following surgery, animals received an injection of antibiotics (oxytetracycline, Terramycine LA®, 1ml/10kg) and an injection of a non-steroidal anti-inflammatory drug (Finadyne®, flumixin megumine, 2ml/50kgs). Animals were followed daily throughout the experiment. Blood samples were collected twice weekly by jugular venipuncture in heparinized tubes. After centrifugation, serum was collected and frozen until assayed for hormones. All animals were killed by decapitation under deep barbiturate anesthesia (Nesdonal®, 5mL). To minimize potential issues linked to time-of-day fluctuation in gene expression (i.e. circadian rhythms) all animals were killed in the early morning (ZT1-4, with ZT0 being the time of lights on, or sunrise).

### Experimental animals & procedures

Experiments were conducted in adult Ile-de-France ewes (3–5 years old; weight 60–80 kg) kept under normal husbandry conditions at the research station of the Institut National de la Recherche Agronomique (Nouzilly, Unité Expérimentale PAO n°1297 (EU0028)). All ewes were ovariectomized (OVX) and estradiol-implanted (E2; 1cm silastic implant) at the beginning of the experiment (three independent experiments; total of n = 74, detailed below). The OVX+E2 model normalizes the level of circulating E2, which uncovers the well-documented central seasonal shift in the negative feedback action of E2 on gonadotropin secretion [17]. In this model, serum levels of the gonadotropins LH and FSH provide a reliable index of the state of the GnRH pulse generator: LH/FSH are low during the non-breeding season, then gradually increase to reach high levels during the breeding season [10,17]. Two groups of intact ewes were also used for a 4^th^ experiment (total of n = 12, n = 6 / group). In total, four independent experiments were performed. For the 1^st^ and 2^nd^ experiments, RNA was extracted from MBH and from the caudal part of the pituitary *pars distalis* (PD; see S1 Fig for details of the dissection procedure). The MBH blocks not only comprise the PT but also include the median eminence, arcuate nucleus, dorsomedian and ventromedian hypothalamic nuclei. The RNA samples from these 2 experiments were used to carry out RT-PCR and qRT-PCR in the current paper and have also been used previously for RNA-seq analysis as detailed further in this M&M (also see [10]). Importantly, many genes with low level of expression (revealed by the number of reads, RPKM), were identified as being differentially expressed by the RNA-seq, which was confirmed by ISH [10]. Furthermore, ISH showed that each candidate gene is specifically expressed in a distinct compartment of the MBH: PT, tanycytes, arcuate nuclei or dorsomedian hypothalamus [10]. Therefore, our approach effectively allows detection of small variations in expression, even for genes expressed to low levels, and by only a specific subset of cells within the MBH.

For the 3^rd^ and 4^th^ experiments ISH was performed on brain sections (see next section and [10]). In the 1^st^ experiment, 18 ewes were kept in an open barn and killed in May (non-breeding season, n = 6), August (when breeding resumes, n = 6) and November (breeding season, n = 6). In the 2^nd^ experiment, ewes (total of n = 24; 3 groups with n = 8/group) were kept indoors under a short photoperiod (SP group, 8.5h of light / day) from December onwards and killed in February, or exposed to a long photoperiod (LP group, 15.5h of light / day) for 3 weeks before culling. A third group had been thyroidectomized (THX) months before the LP exposure (LP-THX group). In animals kept either under prolonged SP or exposed to LP, the hypothalamus drives reproductive arrest, as assessed by LH & FSH levels. In contrast, THX ewes maintain high LH & FSH levels (see [10]). In the 3^rd^ experiment, ISH was performed on brains, which were sampled from ewes maintained outdoors and killed in May and November (total of n = 32; n = 16/group). Half of each group had undergone THX ~5 months prior to the end of the experiment (n = 8/group) while the other half had undergone a sham-operation (n = 8/group). THX specifically blocks the spring transition to non-breeding (see [10]). Finally, in the 4^th^ experiment two groups of intact ewes (total of n = 12; n = 6 / group) were kept outdoors and killed in May and November.

### RNA extraction, standard RT-PCR and qRT-PCR analysis

RNA extraction was performed on each MBH block or PD (see S1 Fig) using TriReagent (Sigma). Concentration and purity of individual samples were determined with Nanodrop 2000 (ThermoScientific) and integrity was checked by standard agarose gel electrophoresis. The same procedure was applied for extraction of RNA from HEK293 cells. For both standard RT-PCR and qRT-PCR, cDNA was synthesised using Omniscript RT kit (Qiagen) and Oligo-dT primers (Eurofins, Germany). Standard PCR was performed with Taq DNA polymerase (Qiagen) using an Applied Biosystems 2720 Thermocycler (Thermofisher). Conditions for PCR were as follows: 95°C for 5 min then 30 cycles: 95°C for 30s / 58°C for 30s / 72°C for 1 min, and a final extension at 72°C for 10 min. PCR reactions were loaded on a 1.5% agarose gel and migrated at 80-110V in Tris-EDTA-Acetic acid (TEA) buffer for ~30min.

For qRT-PCR, the optimal cDNA dilution and calibration curves for *Vegfa* and *VegfR2* primer pairs (Eurofins) were established using cDNA synthesized from an equimolar mix of individual RNA from each experiment. As a negative control, the same mix with water instead of RT was prepared. Quantitative PCR was performed using CFX-96 Real-Time PCR Detection System (Bio-Rad) and Sso Adv Universal SYBR Green Supermix (Bio-Rad). All samples (unknown, standard curves) were assessed in triplicate and Rplp0 (ribosomal subunit P0, a.k.a 36B4) was used as a housekeeping gene; Rlpl0 expression did not display any statistical differences between groups (data not shown). The quantification of mRNA level was obtained by the 2^-DCT^ method and data are presented as fold-increase compared to the condition with the lowest expression level. For both standard PCR and qPCR, the identity of amplicons was validated by cloning (pGEMT easy vector, Promega) and Sanger sequencing (Eurofins Sequencing services, Germany).

### Analysis of Illumina RNA-seq datasets

We analyzed two independent RNA-seq datasets generated by Illumina sequencing. The first dataset was generated using the same MBH RNA samples which are used here to perform qRT-PCR as well as RT-PCR. All details regarding the procedures, thorough validation and outcomes of these RNA-seq experiments (comparisons between May/August/November = Expt 1 under M&M and comparisons between SP/LP/LP-THX = Expt 2 under M&M) have recently been published [10]. The second dataset was independently generated using RNA extracted from the PT of castrated rams (see details in [9]). First, the data were re-mapped to Oar_V4.0 (ftp://ftp.ncbi.nlm.nih.gov/genomes/genbank/vertebrate_mammalian/Ovis_aries/latest_assembly_versions/GCA_000298735.2_Oar_v4.0) using STAR RNA-seq aligner and standard mapping options [18]. STAR allows the identification of *de novo* and annotated splice junctions and the algorithm uses quality scores to increase its efficacy. Annotation from ftp://ftp.ncbi.nih.gov/Ovis_aries/GFF/ref_Oar_v4.0_top_level.gff3.gz were used to define known junctions. Novel junctions are identified by STAR based on uniquely mapping reads. Using a custom perl script the junctions were filtered for the *Vegfa* gene. The data represent all the junctions identified with >10 reads.

### In situ hybridization

Hypothalamic blocks for *in situ* hybridisation were cut into 20 micrometer sections using a cryostat (CryoStar NX70, ThermoScientific) and thaw-mounted onto SuperFrost Plus slides (ThermoScientific). The radioactive *Vegfa* cRNA riboprobe was prepared by plasmid linearisation and *in vitro* transcription (Riboprobe System, Promega) including ^35^S-UTP (Perkin-Elmer). The probe was purified with Illustra Probe Quant G50 micro-columns (Fisher) and counted with a liquid scintillation counter (Tri-Carb 2900TR, Packard). Slides were post-fixed at 4°C for 20 min in 4% PFA, 0.1 M PB, rinsed with 0.1 M PB (2 X 5min), acetylated with 3.75% v/v of acetic anhydride in 0.1 TEA, 0.05 N NaOH (10min) and finally rinsed with 0.1 M PB (2 X 5min). Slides were then dehydrated through graded ethanol solutions (50%, 70%, 95% and 100%; 3min each) and dried under vacuum for 60 min. Sections were hybridized overnight at 58°C with 10^6^ cpm of probe per slide in hybridization buffer (50% deionized formamide, 10% dextran sulfate, 1 X Denhardt’s solution, 300 mM NaCl, 10 mM Tris, 10 mM DTT, 1 mM EDTA, 500 micrograms/ml tRNA). Sections were then rinsed in 4 X SSC (3 X 5 min) and subjected to RNase-A digestion (20 micrograms/ml) in a buffer containing 500 mM NaCl, 1 mM Tris, 1 mM EDTA for 30 min at 37°C. Stringency washes in SSC (with 1mM DTT) were performed to remove non-specific probe hybridisation: 2 X SSC (2 X 5 min), 1 X SSC (10 min), 0.5 X SSC (10 min), 0.1 X SSC (30 min at 60°C), 0.1 X SSC (5 min). Slides were then dehydrated through graded ethanol solutions (50%, 70%, 95% and 100%; 3min each), dried under vacuum for 60 min and exposed for a week to an autoradiographic film (BioMax MR, Kodak). Films were scanned on a transmittance image scanner (Amersham, UK) along with a calibrated optical density (OD) transmission step wedge (Stouffer, USA). Calibrated Integrated OD measurements of gene expression in the PT were performed using ImageJ software.

### Data analysis

Data were analysed using GraphPad Prism and are reported as mean ± sem. qRT-PCR data were analyzed by 1-way ANOVA using time or treatment as variables. The post-hoc Tukey test was used for mulitple comparisons. ISH data were analysed by either t-test or 2-way ANOVA using time and treatment as variables. p<0.05 was considered significant.

## Results

A complete list of the primers used in this study is provided in Table 1.

**Table 1:** List of primers.

Primer (strand)	5’ -> 3’ sequence	Function
**Vegfa primers**		
O12I (+)	ACCCTGGTGGACATCTTCC	Initial PCR screening
O14I (+)	CACCAAAGCCAGCACATAGG	Initial PCR screening & ISH probe
O15I (-)	AAGTGCTCTGCGCACAGC	Initial PCR screening & ISH probe
O16I (-)	AGGAACTGTGCTGGGTCAC	Initial PCR screening
O17I (+)	GCTCTCTTGGGTGCATTGG	qPCR (Exon 1)
O20I (-)	TCAGTGGGCACACACTCCAG	qPCR (Exon 3)
O21I (-)	CTTTCCTGGTGAG**ACAT**TTTTC	5’ exon 8b / 3’ anchor in exon 5
O22I (-)	TCCTGGTGAG**ACAT**CTGCA	5’ exon 8b / 3’ anchor in exon 7 / 4 / 3
O23I (-)	TCCTGGTGAG**ACAT**CTGGT	5’ exon 8b / 3’ anchor in exon 8 (positive ctrl)
O61I (-)	CTGGTGAG**ACAT**CTGCAAG	5’ exon 8b / 7 bp anchor exon 7 / 4 /3
O62I (-)	TGGTGAG**ACAT**CTGCAAGTA	5’ exon 8b / 9 bp anchor exon 7 / 4 /3
O63I (-)	GTGAG**ACAT**CTGCAAGTACG	5’ exon 8b / 11 bp anchor exon 7 / 4 /3
O64I (-)	GGTGAAACTCTGCAAGTACG	5’ = O63I scrambled / 11 bp anchor exon 7 / 4 /3
O65I (-)	CTTCCTCCACTGCAAGTACG	5’ exon 8 (random seq) / 11 bp anchor exon 7 / 4 /3
O66I (-)	AAACCCTGACTGCAAGTACG	5’ exon 8 (random seq) / 11 bp anchor exon 7 / 4 /3
O67I (-)	GTCAGTCTTCTGCAAGTACG	5’ exon 8-8b (random seq) / 11 bp anchor exon 7 / 4 /3
O68I (-)	TGTCAGGTTCTGCAAGTACG	5’ exon 8-8b (random seq) / 11 bp anchor exon 7 / 4 /3
O73I (-)	**ACAT**CTGCAAGTACG	5’ exon 8-8b (junction) / 11 bp anchor exon 7 / 4 /3
O74I (-)	**CAT**CTGCAAGTACG	5’ exon 8-8b (junction) / 11 bp anchor exon 7 / 4 /3
O75I (-)	**AT**CTGCAAGTACG	5’ exon 8-8b (junction) / 11 bp anchor exon 7 / 4 /3
O76I (-)	**T**CTGCAAGTACG	5’ exon 8-8b (junction) / 11 bp anchor exon 7 / 4 /3
O77I (-)	CACTC**ACAT**CTGCAAGTACG	O63I but reverse seq of 5’ exon 8b specific
O78I (-)	GGGGG**ACAT**CTGCAAGTACG	O63I but G stretch instead of seq 5’ exon 8b specific
O79I (-)	AAAAA**ACAT**CTGCAAGTACG	O63I but A stretch instead of seq 5’ exon 8b specific
O80I (-)	GTGAG**T****CAT**CTGCAAGTACG	O63I with one mismatch
O81I (-)	GTGAG**A****G****AT**CTGCAAGTACG	O63I with one mismatch = hVEGF165b specific primer
O82I (-)	GTGAG**TG****AT**CTGCAAGTACG	O63I with two mismatches
O90I (-)	CACTC**GG****AT**CTGCAAGTACG	O63I two mismatches + rev. seq of 5’ exon 8b specific
**VegfR2 (Kdr) primers**		
O25I (+)	GGGACTCTCTCTGCCTACCT	qPCR
O27I (-)	ATACCACTGTCCGTCTGGCT	qPCR

### Seasonal expression of *Vegfa* and *VegfR2* in the ovine MBH and *pars distalis* (PD)

Quantitative RT-PCR or *in situ* hybridization (ISH) were used to determine level and profile of expression of *Vegfa* and its cognate receptor *VegfR2* in the MBH and PD in 4 experiments.

**Fig 1:**
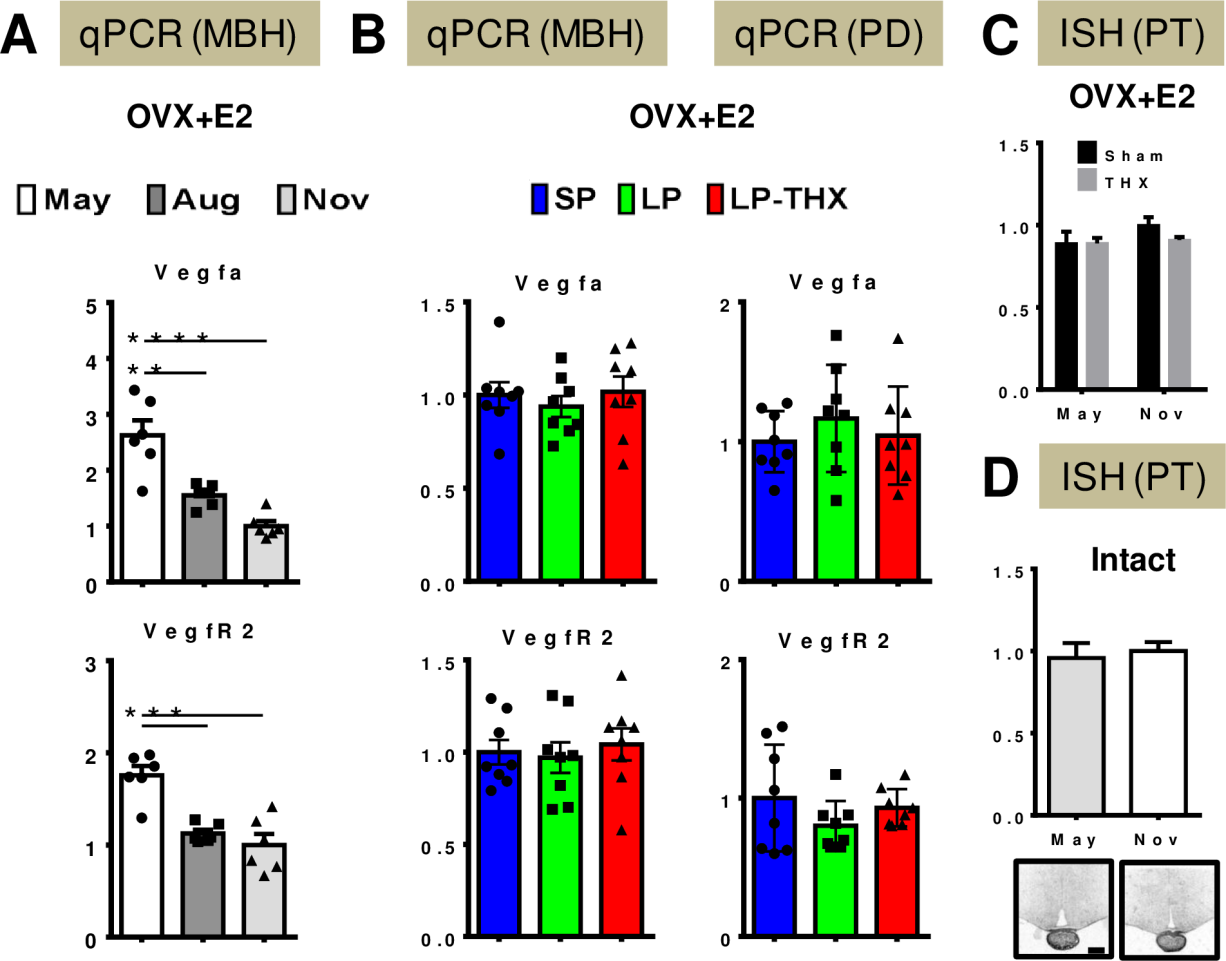
Seasonal expression of *Vegfa* and *VegfR2* in the ovine MBH and pituitary. All values on the y-axis are normalized relative levels of expression (qPCR) or optical density (ISH). A/ 1^st^ Experiment: qRT-PCR for *Vegfa* and *VegfR2* on MBH cDNA samples from OVX+E2 ewes maintained under natural conditions and culled in May, August and November. Post-hoc Tukey test: **p<0.01, ***p<0.005 & ****p<0.001. B/ 2^nd^ Experiment: qRT-PCR for *Vegfa* and *VegfR2* on MBH (left column) and PD (right column) cDNA samples from OVX+E2 ewes, which had been kept indoors under prolonged SP or exposed to 3 weeks of LP. A group of LP-exposed ewes had been THX months before photoperiodic transfer (LP-THX). C/ 3^rd^ Experiment: ISH for *Vegfa* on coronal brain sections at the level of the caudal PT / infundibulum region. Sham-operated and THX ewes (OVX+E2 model) were sampled in May and November and quantification was performed in the PT. D/ 4^th^ Experiment: ISH for *Vegfa* on coronal brain sections at the level of the caudal PT / infundibulum region. Intact ewes were sampled in May and November and quantification was performed in the PT. Representative autoradiograms are shown (scale bar = 2mm).

In the 1^st^ experiment qRT-PCR was used to evaluate expression of *Vegfa* and *VegfR2* in MBH tissue blocks collected in May (non-breeding season), August (breeding season resumes) and November (breeding season), with n = 6 for each group. Such a block comprises the PT, median eminence, arcuate nucleus, dorsomedian and ventromedian hypothalamic nuclei. Details of the tissue sampling are provided in S1 Fig. Both *Vegfa* and *VegfR2* showed a ~2-fold decrease in expression between May and August and remained low through to November (Fig 1A).

In the 2^nd^ experiment, we assessed *Vegfa* and *VegfR2* expression in the MBH block and in the PD of the pituitary of 3 groups of ewes (n = 8 / group) kept under SP or exposed to an acute LP stimulus (both sham-operated and THX): neither of these genes showed any difference in expression levels amongst tissues or groups (Fig 1B).

In the 3^rd^ experiment, ISH was performed on hypothalamic sections from ewes also sampled in May and November (n = 16/group). In addition, half of each group had undergone THX ~5 months prior to the end of experiment. The use of ISH on coronal sections showed strong *Vegfa* expression in the PT with no difference in relative levels amongst groups (Fig 1C).

In the 4^th^ experiment intact ewes were killed either in May or in November. Here again, ISH on coronal sections showed strong *Vegfa* expression in the PT, which was not different between May and November as revealed by quantification on autoradiograms (Fig 1D).

In conclusion, *Vegfa* and *VegfR2* are expressed in the MBH and PD of the ewe. Both *Vegfa* and *VegfR2* display moderate seasonal modulation in the MBH, with higher levels during the non-breeding season. Within the MBH, the PT appears to be the main site of *Vegfa* expression, with no obvious correlation between relative levels and the reproductive status. In these experiments, the PCR primers for qRT-PCR and the probe used for ISH were designed to detect all *Vegfa* transcripts; i.e. they would not allow discrimination between *Vegfaxxx* and *Vegfaxxxb* isoforms. We therefore designed approaches to specifically investigate expression of *Vegfaxxx* / *Vegfaxxxb* splice variants across seasons and physiological states.

### Background: Organization of the ovine *Vegfa* gene

**Fig 2:**
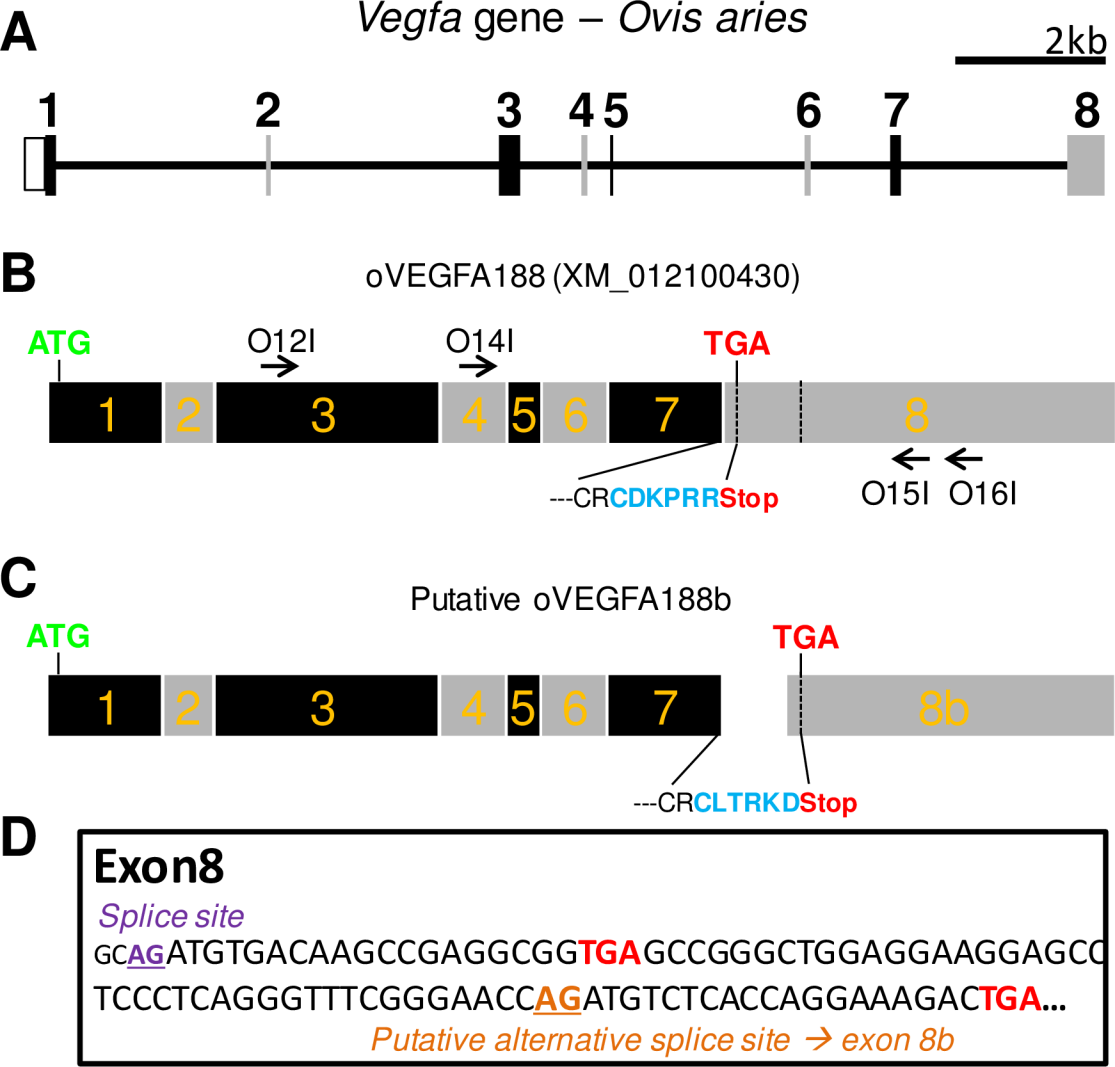
Organization of the ovine *Vegfa* gene. A/ Schematic of the ovine *Vegfa* gene locus. The eight exons are labeled and represented by alternating black and gray boxes. B/ Schematic of the longest transcript, which includes the 8 exons and is predicted to yield a mature protein of 188 amino acid, oVEGFA188. The location of both start (ATG) and stop (TGA) codons, the positions of upstream (O12I & O14I) and downstream (O15I & O16I) PCR primers for non-isoform specific PCR (see Fig 3) and the amino acids sequence of the resulting C-Term are provided. C/ Schematic of the putative *oVegfa188b* alternatively spliced transcript, which would use a cryptic acceptor site in exon 8, therefore renamed exon 8b. The location of both start (ATG) and stop (TGA) codons are indicated. Note that only the C-term of VEGFA188 and VEGFA188b would differ. D/ Nucleotide sequence of the proximal part of ovine exon 8. The splice sites for both isoforms and the respective stop-codons are indicated. The lower case indicates the intronic sequence (for the *Vegfaxxx* transcripts).

The ovine *Vegfa* locus is located on chromosome 20, spans ~14kb and comprises 8 exons (Fig 2A; GenBank: NC_019477; Gene ID: 443103). Automated computational analysis predicts 4 transcripts (see S2 Fig): (i) XM_012100430, which comprises all 8 exons and encodes a mature protein of 188 amino acids (VEGFA188; Fig 2B); (ii) XM_012100431, which contains all 8 exons but uses an alternatively spliced form of exon 6, which keeps the ORF and yields VEGFA182; (iii) XM_012100432, in which exon 6 skipping leads to VEGFA164 and (iv) NM_001025110, in which both exons 6 and 7 are skipped, thereby leading to VEGFA120.

The existence of one or several additional transcripts, *Vegfaxxxb*, has been proposed in sheep [11]. Such transcript(s) would comprise an alternatively spliced exon 8 (exon 8b), due to the use of a cryptic acceptor splice site, located downstream of the known acceptor site (Fig 2B, 2C & 2D). Such transcripts would therefore encode VEGFAxxxb proteins with a CLTRKD motif on their C-Term, rather than the CDKPRR motif (Fig 2B & 2C). We first aimed at the identification of splicing variants of the *Vegfa* gene using a RT-PCR methodology suitable for amplification of both *Vegfaxxx* and *Vegfaxxxb* transcripts.

### Identification of ovine *Vegfa* transcripts by standard PCR reveals 5 *Vegfaxxx* isoforms

We first performed standard RT-PCR using an equimolar mix of RNA extracted from the 18 MBH blocks from OVX+E2 ewes of the 1^st^ experiment described above (May/August/November). Therefore, our cDNA preparation is expected to contain a representative set of seasonal *Vegfa* transcripts, should there be variation in their relative proportions throughout the year. As can be deduced from Fig 2, *Vegfaxxxb* PCR products would be expected to migrate approximately 66bp below their *Vegfaxxx* counterparts.

**Fig 3:**
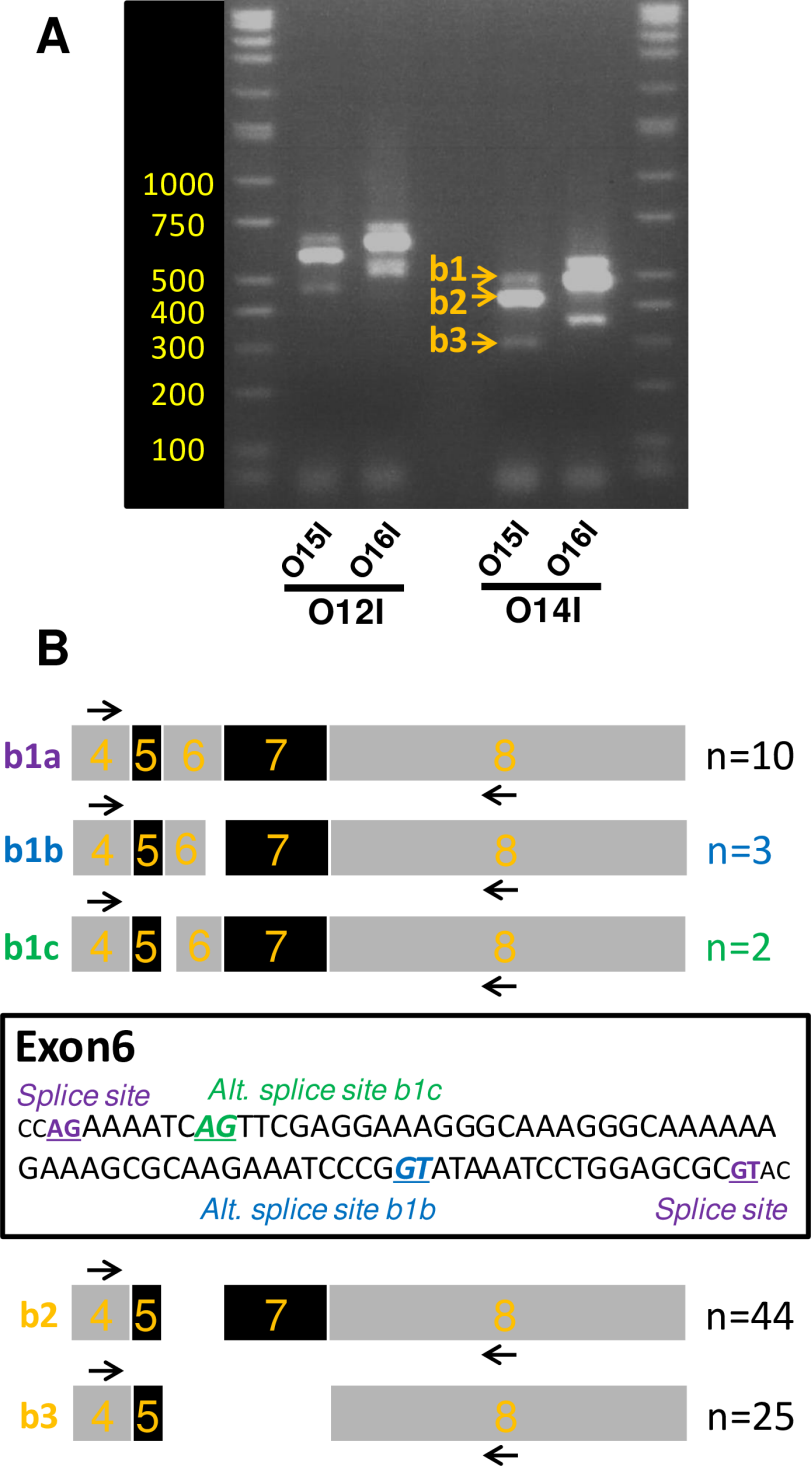
Identification of ovine *Vegfa* transcripts by standard PCR using primers O14I/O15I: No evidence for *Vegfaxxxb* isoforms. A/ Picture obtained after agarose gel electrophoresis of *Vegfa* PCR products. PCR was performed on a cDNA mix obtained from MBH of ewes sampled in May, August and November. The primers were designed to amplify both Vegfaxxx and Vegfaxxxb products. A typical “3-bands pattern” was obtained for all 4 primer combinations. The 3 bands, labeled b1, b2 and b3 (arrows), were individually extracted on gel for the primer combination O14I/O15I. B/ Schematics summarizing the results of Sanger sequencing after cloning of PCR products contained in b1, b2 and b3 (location of primers O14I/O15I is provided). Five distinct amplicons were identified: 3 for b1 (labeled b1a, b1b and b1c) and a single product for both b2 and b3. The number of clones sequenced for each amplicon is indicated on the right. An annotated sequence of ovine exon 6 is provided and the alternative splice sites used to generate the transcripts b1b and b1c are identified. Note that 0/84 clones included the putative exon 8b.

We performed four different PCR reactions using combinations of two upstream primers, located within exon 3 and exon 4, respectively (O12I & O14I, Fig 2B, Fig 3A, Table 1) and two downstream primers, both located in exon 8, >120bp downstream of the predicted alternative splice site yielding exon 8b (O15I & O16I, Fig 2B, Fig 3A, Table 1). As assessed by agarose gel electrophoresis, all four PCR combinations yielded a distinctive “3-bands pattern” (b1, b2 and b3; Fig 3A), consistent with the existence of at least three different transcripts for *Vegfa*. The three bands obtained with the O14I/O15I primer pair (Fig 3A) were extracted from the gel, cloned and sequenced (Fig 3B).

A total of 84 clones were sequenced: 15 clones for b1, 44 clones for b2 and 25 clones for b3 (Fig 3B). This revealed that band1 comprises at least three distinct transcripts: b1a (500bp; 10 clones) corresponds to XM_012100430; b1b (482bp; 3 clones) corresponds to XM_012100431; b1c corresponds to a novel transcript (492bp; 2 clones) which uses a cryptic acceptor site at the beginning of exon 6 thereby removing 8bp. As a consequence, this b1c transcript would encode a truncated protein product. The 44 clones for Band 2 represent a single transcript, which corresponds to XM_012100432 (428bp, exon 6 skipped). Finally, the 25 clones for Band 3 also represent a single transcript, which corresponds to NM_001025110 (296bp, both exons 6 and 7 skipped). All clones sequenced and transcripts identified correspond to the usage of the “classical” splice site for exon 8.

### Specifically searching for *Vegfaxxxb* transcripts with isoform-specific PCR primers

We reasoned that failure to identify *Vegfaxxxb* transcripts might be due to a low level of expression and/or a very tight, season-specific, expression pattern. Therefore, we used cDNA pools from each group of animals of the 1^st^ experiment (i.e. May, August and November, n = 6/condition; Fig 1A) rather than a cDNA pool generated with a RNA mix from all 18 animals. In a strategy broadly similar to that used by Harris *et al*. for mouse and human tissues [15], we designed two *Vegfaxxxb*-specific downstream primers: O21I comprises the last 5 bases of exon 5 and the first 17 bases immediately downstream of the splice site for putative exon 8b while O22I comprises the last 5 bases of exon 7 and the 14 bases immediately downstream of the splice site for putative exon 8b. The last 5 bases of exon 7 (5’-TGCAG-3’) are identical to the last 5 bases of exon 4 and 3 (see Table 1).

**Fig 4:**
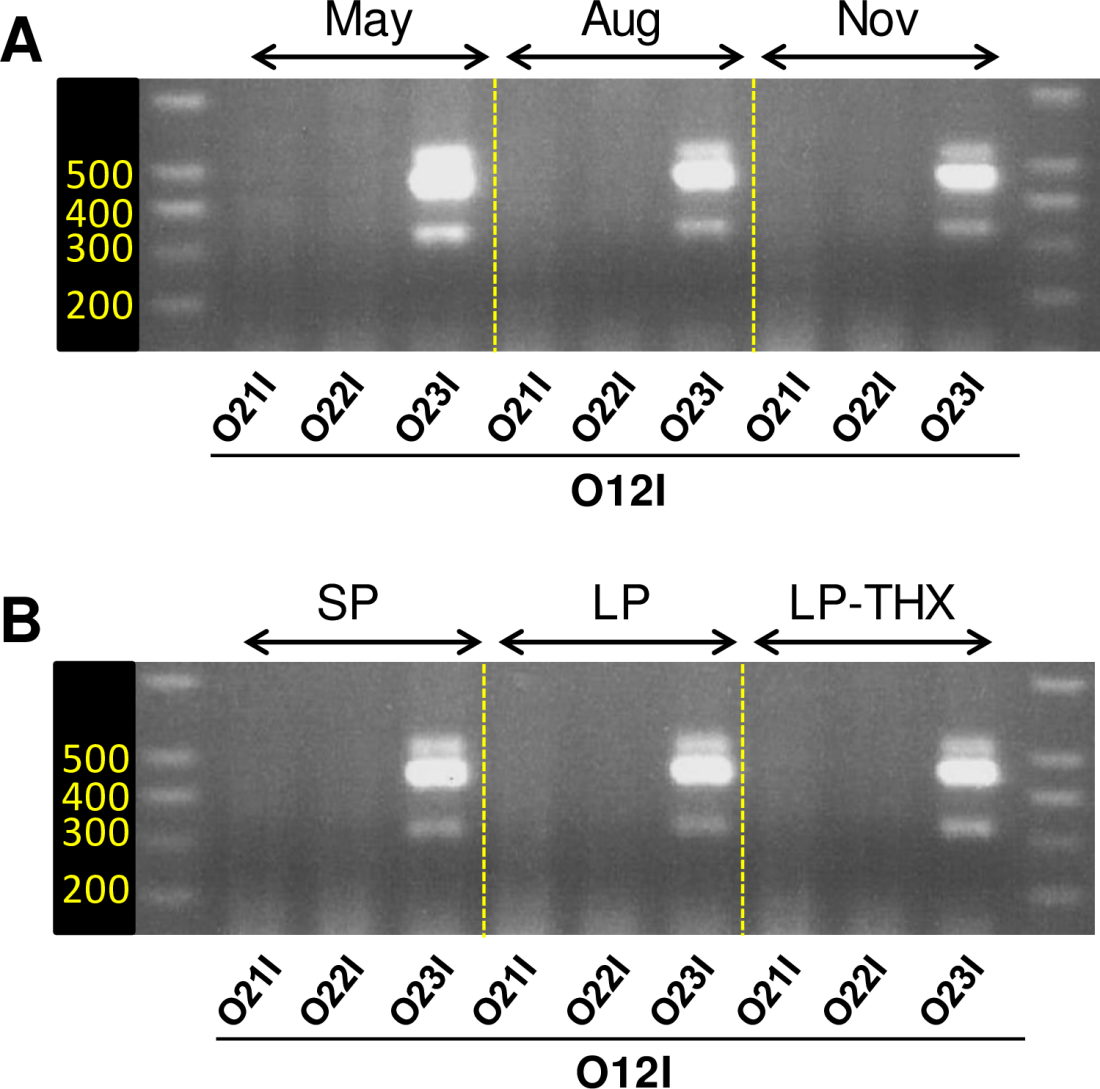
Searching for *Vegfaxxxb* transcripts with isoform-specific PCR primers. A/ Standard PCR / agarose gel electrophoresis was performed using MBH extracts from ewes culled across seasons (same samples used for qPCR data in Fig 1). O21I and O22I are Vegfaxxxb-specific primers while O23I spans the putative splice site for exon 8b (and is therefore a Vegfaxxx-specific primer). B/ Standard PCR / agarose gel electrophoresis was performed using MBH extracts from ewes culled under SP or after a photoperiodic transfer to LP (intact and THX ewes; same samples used for qPCR data in Fig 1).

Therefore, O21I is designed to amplify *Vegfaxxxb* transcripts that lack both exons 6 and 7 while O22I would amplify transcripts that include exon 7. Based on findings for *Vegfaxxx*, this primer design encompasses the complete set of putative *Vegfaxxxb* transcripts. We designed an additional downstream primer (O23I), which bridges the putative splice junction for exon 8b. This primer would amplify all *Vegfaxxx* isoforms and therefore serves as a positive control. These primers were used in combination with the upstream primer O12I for all PCR reactions. Whether performed with cDNA from animals culled in May, August or November, PCR reactions using *Vegfaxxxb*-specific primers (O21I & O22I) did not yield any PCR product (Fig 4A). In contrast, PCR with the O12I/O23I pair resulted in a very robust “3-bands pattern” of amplification.

The same PCR reactions were also performed using cDNA synthesized from MBH RNA extracts from the 2^nd^ experiment (see Fig 1B). Here again, the O12I/O23I pair led to robust PCR amplification of the “3-bands pattern” while the *Vegfaxxxb*-specific primers O21I & O22I did not yield any PCR product (Fig 4B).

### Analysis of RNA-seq datasets: Strong support for the existence of *Vegfaxxx* splice variants but not for *Vegfaxxxb* isoforms

We next analyzed two independent RNA-seq datasets generated by Illumina sequencing. A summary of the results is presented in Table 2 and S2 Fig.

**Table 2:** RNA-seq identifies intron-spanning, uniquely mapping reads at the *Vegfa* gene.

	Intron position				Uniquely mapping reads (UMR)	
***Ovis aries Vegfa* gene NC_019477.2**	1^st^ base	Last base	Exon junction	# band (Fig 2)	Lomet *et al*. [10]	Wood *et al*. [9]
	17291151	17293916	1*2		546	222
	17293966	17297121	2*3		864	237
	17297319	17298092	3*4		1015	285
	17298170	17298492	4*5		2374	410
	17298523	17301128	5*6		224	34
	17298523	17302286	5*7	b2	1631	316
	17298523	17304586	5*8	b3	331	77
	17301201	17302286	6*7	b1a	361	31
	17301183	17302286	6*7	b1b	147	17
	17302419	17304586	7*8		2082	203
**Reads : Total**					**9575**	**1832**
**Reads defining start of canonical exon 8**					**2693**	
**Reads defining start of putative exon 8b**					**0**	

Collectively, the two datasets yielded 11407 intron-spanning uniquely mapping reads (UMR), which cover all known exonic junctions of the *Vegfa* gene (see S2 Fig). The data provide strong support for the existence of all splice-variants identified previously, with the exception of the rare b1c transcript (see 5^th^ column of Table 2 and Fig 2). Importantly, 408 of these UMR cover the junction between exon 5 and exon 8 (highlighted in grey) while 2285 cover the junction between exon 7 and exon 8 (also highlighted in grey). Thus, a total of 2693 UMR support the existence of the canonical splice site for exon 8. In contrast, no UMR supports the existence of the alternative splice site yielding the putative exon 8b.

To determine whether prior investigations identified “*Vegfaxxxb*-like” PCR products due to methodological issues in primer design we aimed to define the minimal primer requirements that lead to amplification of “*Vegfaxxxb*-like” PCR products.

### How many bases complementary to exon 7 to yield “*Vegfaxxxb*-like” PCR products?

We focused our analysis on transcripts that would include exon 7, since these represent the majority of *Vegfa* transcripts (b1 and b2, see Fig 3B and Table 2). We first wished to define how many bases specific to exon 7 are required to yield “*Vegfaxxxb*-like” PCR products. As demonstrated by the use of O22I (Fig 4), 5 bases is not enough. Using an approach similar to that used by Harris et al [15], we designed downstream primers with a 3’ end that includes 7, 9 or 11 bases complementary to the 3’ end of exon 7 (O61I, O62I & O63I), while adjusting the length of the “exon 8b-specific” 5’ part of the primer to maintain similar melting temperatures (Tm) across primers.

**Fig 5:**
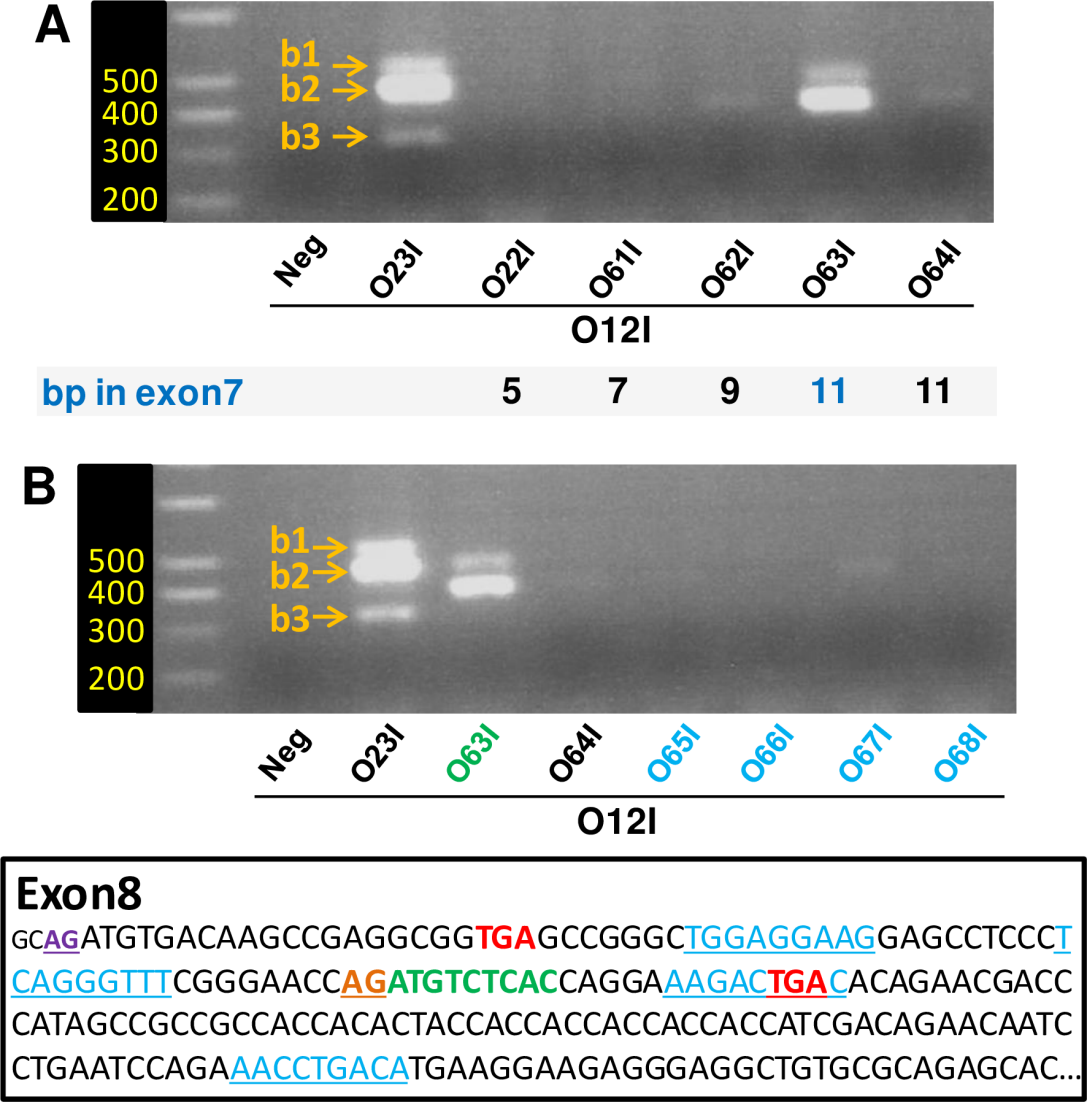
Defining features of an error-prone primer yielding “*Vegfaxxxb*-like” products. Standard PCR / agarose gel electrophoresis was performed using MBH extracts from ewes culled across seasons. A/ 20 bases downstream primers including a variable number of bases complementary to exon 7 were tested. O23I was used a positive control. A minimum of 11 bases complementary to exon 7 was required to yield “*Vegfaxxxb*-like” PCR products. The sequence of the 9 bases complementary to putative exon 8b also matters (9 bases scrambled primer O64I, see text). Neg: cDNA was omitted from the PCR reaction. B/ PCR was performed with various 20 bases downstream primers, which all contain the 3’end with the 11 bases complementary to exon 7 and a 5’end with 9 bases stretches complementary to variable sequences within exon 8. Bottom panel provides the sequence of exon 8. Locations of the 9 bases stretches used for designing 5’ends of O65I-O68I are underlined in blue. A PCR product was obtained only with the “exon 8b-specific” O63I primer; the 9 bases stretch of this primer is indicated in bold green. O23I was used a positive control.

PCR with O23I (positive control, see Fig 4) led to the typical “3-band pattern” (Fig 5A), while PCR with O22I (negative control, see Fig 4), O61I and O62I yielded at best very faint PCR amplification. In contrast, PCR with O63I resulted in robust amplification of “*Vegfaxxxb*-like” PCR products, corresponding to b1 and b2. We therefore surmised that the 11 bases at the 3’end, which hybridize to exon 7, suffice to get robust PCR amplification. Accordingly, we designed O64I, which retains these 11 bases while the other 9 bases of O63I are scrambled. Interestingly, very little PCR amplification was obtained with O64I compared to O63I (Fig 5A). In sum, to yield “*Vegfaxxxb*-like” PCR products, the “*Vegfaxxxb*-specific” downstream primer must include a minimum of 11 bases complementary to exon 7. Additionally, the 9 bases of the “exon 8b-specific” 5’ part of the primer also contain feature(s) required for efficient PCR amplification.

### Critical feature(s) of the 9 bases of the “exon 8b-specific”: Ruling out cooperativity

We first tested the hypothesis that the 9 bases of O63I are involved in some form of cooperativity: since these 9 bases are complementary to the *Vegfa* mRNA sequence (contrary to the non-specific scrambled sequence of O64I) they might hybridize to the *Vegfa* transcript, thereby bringing the 3’end of the primer closer to its target sequence (i.e. 3’ end of exon7), which may indirectly promote PCR. To test this hypothesis, we designed four primers (O65I, O66I, O67I and O68I), which include a stretch of 9 bases complementary to sequences contained within exon 8-8b (Fig 5B, sequences in blue and underlined in bottom panel). We kept the AT/GC content of these 9 bases identical to that of O63I (Fig 5B, sequence in green and bold in bottom panel). As seen in Fig 5B, none of these primers led to any significant PCR amplification compared to O63I. We therefore ruled out some form of “cooperativity” as a potential explanation for the amplification specifically seen when using O63I.

### Critical feature(s) of the 9 bases of the “exon 8b-specific”: Exon 8 and putative exon 8b share a common 4 bases motif at their 5’ end

**Fig 6:**
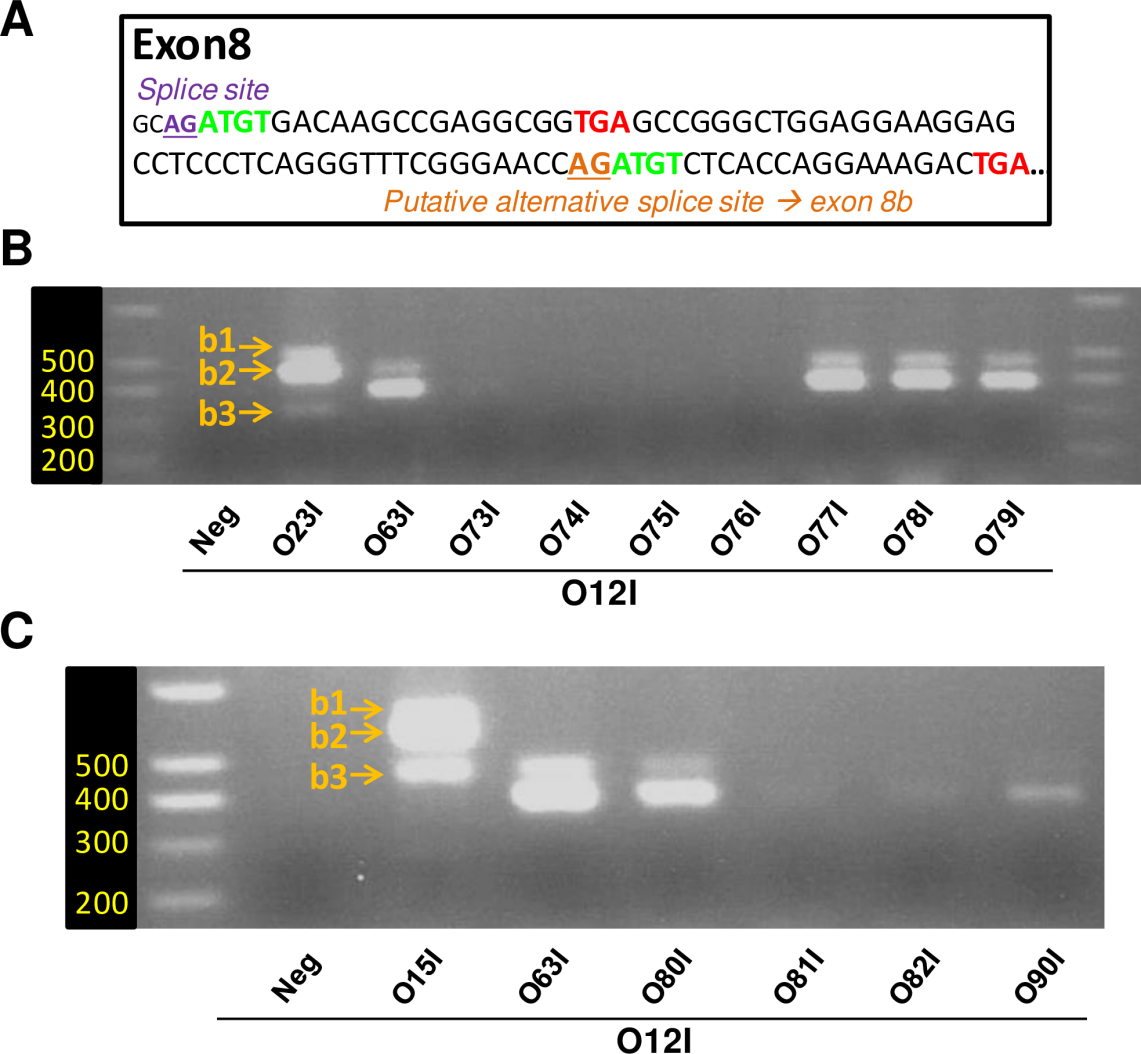
Exon 8 and putative exon 8b share a common 4 bases motif at their 5’ end. A/ Nucleotide sequence of ovine exon 8, splice sites are indicated. Both exon 8 and exon 8b would start with the same 4 bases motif ATGT, in green and bold. B/ Standard PCR / agarose gel electrophoresis was performed using MBH extracts from ewes culled across seasons. The use of downstream primers, which all contain the 3’end with the 11 bases complementary to exon 7 and a 5’end that includes these 4 bases or less (O73I-O76I), did not yield any PCR products. However downstream primers, which all contain the 3’end with the 11 bases complementary to exon 7, along with a 5’end that includes these 4 bases preceeded by a random stretch of 5 bases (O77I-O79I), all yielded robust amplification. C/ Single or double point mutation were made to the ATGT motif in the backbone of “exon 8b-specific” O63I primer. Note that a single point mutation (ATCT, O81I) is enough to blunt PCR (see text for details).

A closer look at exon 8 sequence revealed that the four bases immediately following the splice sites of exon 8 and putative exon 8b are identical: spliced transcripts would both start with the same ATGT motif (bold green, Fig 6A). As a consequence, exon O63I not only comprises 11 bases complementary to exon 7 but also includes 4 bases, which would correspond to either exon 8 or putative exon 8b. Consequently, the 15 bases at the 3’ end of this primer would be complementary to either *Vegfaxxx* or *Vegfaxxxb* transcripts.

We reasoned that these 4 bases might explain why O63I leads to PCR amplification while other primers do not. As a first step, we designed primers O73I, O74I, O75I and O76I, whose sequence corresponds to the last 15, 14, 13 and 12 bases of the 3’end, respectively. As seen in Fig 6B, no PCR amplification was obtained with these primers. This was not unexpected since these PCR primers are very short and therefore have quite a low theoretical Tm: 44°C for O73I, down to 36°C for O76I (using G/C: 4°C, A/T: 2°C). We hypothesized that the 5 extra-bases missing from O73I are not required for specificity (i.e. primer hybridization) but that their mere presence increases the Tm of the primer, which allows PCR to proceed. If true, addition of virtually any 5 bases upstream of the 15 bases stretch might support PCR amplification. To test this, we designed three primers derived from O73I, with variable stretches of 5 bases at their 5’ end: O77I, with a 5’-CACTC- motif (mirror of the 5-GTGAG- motif of O63I), O78I with a 5’-GGGGG- stretch and O79I with a 5’-AAAAA- stretch. All three primers were as efficient as O63I (Fig 6B). This unambiguously validates our hypothesis that the extra bases on the 5’end do not confer specificity but are merely required to increase Tm.

Next, we wondered if, in the context of a 20 bases primer, all 4 bases of the ATGT motif (5’-ACAT for the downstream primer) are actually required for PCR amplification. To test this, we designed primers with point mutations. As seen before, robust PCR amplification was observed using O15I and O63I as positive controls (Fig 6C). Interestingly, PCR also worked with O80I, in which the last base of the motif is mutated to yield a 5’-TCAT motif. In contrast, mutating the third (5’-AGAT, O81I) or both the third and fourth bases of the motif (5’-TGAT, O82I) severely impaired PCR. Interestingly, a primer with a 5’-CACTCGGAT motif (O90I), which spares only the first two bases of exon 8 led to substantial PCR amplification.

**Fig 7:**
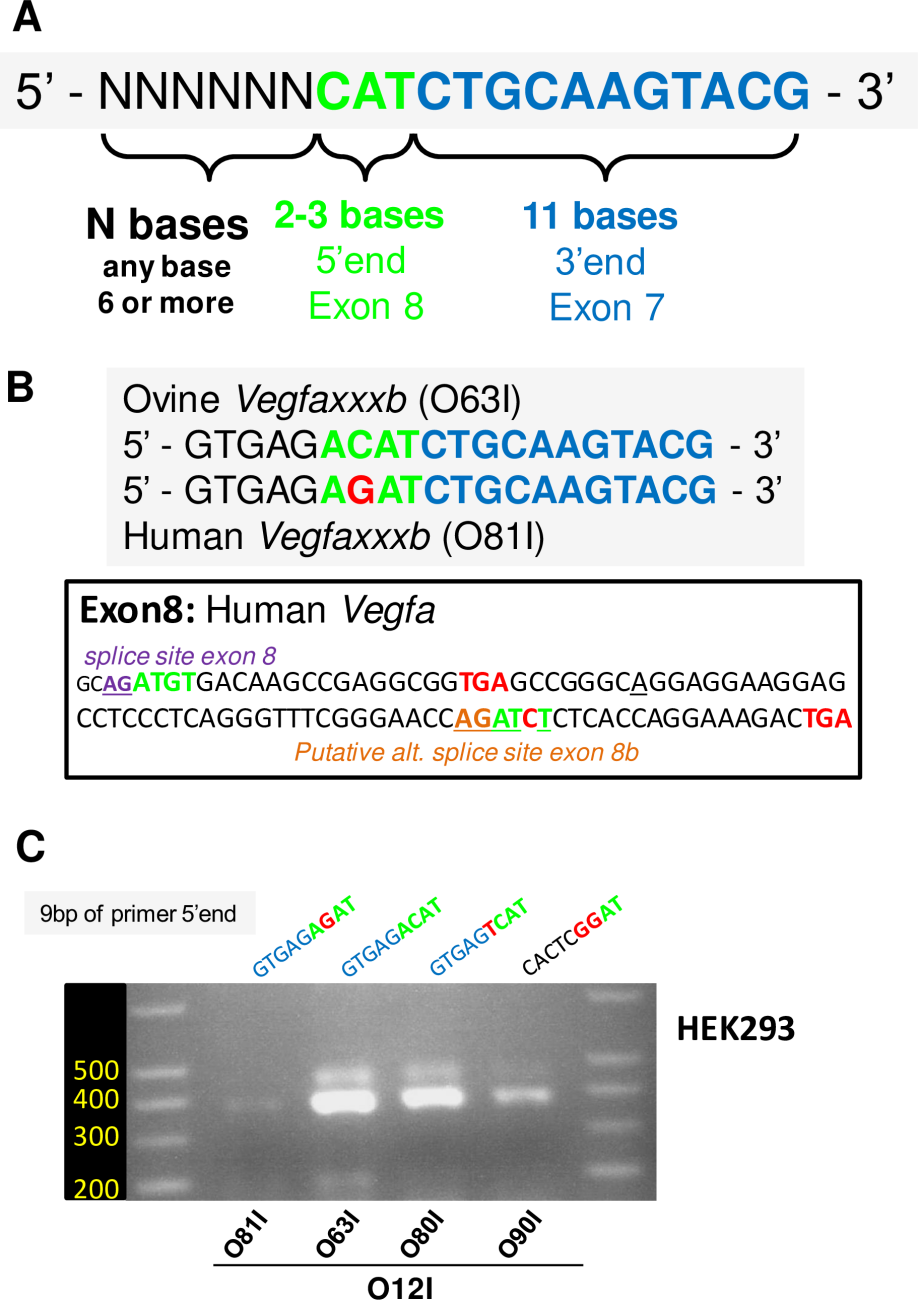
*Vegfaxxxb* transcripts as PCR artefacts: Extending findings to the human-derived HEK293 cell line. A/ Proposed sequence for the prototypical “*Vegfaxxxb*-specific” PCR primer. Note that beyond the 11 bases complementary to exon 7 and a minimum of 2–3 bases complementary to exon 8, the sequence at the 5’end of the primer does not confer specificity. However, this 5’ tail is mandatory as it increases the Tm of the primer. B/ Sequence alignments of the ovine “*Vegfaxxxb*-specific” PCR primer O63I, and the human “*Vegfaxxxb*-specific” PCR primer O81I as defined by Bates and coll. [19]. Note that the two sequences differ only by a single nucleotide, located in the 4 bases motif. The sequence of the proximal part of human *Vegfa* exon 8 is provided. C/ PCR on cDNA samples obtained from HEK293 cells. The sequences of the 9 bases stretches of the 5’end of the downstream primers are provided above the gel picture to facilitate interpretation. The human “*Vegfaxxxb*-specific” primer O81I yielded a faint signal compared to O63I and O80I, which are either single or double point mutations of O81I - but respectively spare all 4 bases and 3 bases of the ATGT motif for exon 8. Note that even a primer bearing an unrelated 7 bases stretch at its 5’ end (O90I) could outperform O81I.

We conclude that amplification of “*Vegfaxxxb*-like” PCR products may be obtained through primer design. We identified the minimal features of an error-prone PCR primer (Fig 7A): a 3’end with 11 bases complementary to exon 7, preceded by a minimum of 2–3 bases complementary to either exon 8 or “putative exon 8b” and the addition of at least 5–6 bases (any bases) at the 5’end. The 5’ end appears to be solely required to increase Tm, which allows PCR to proceed.

### *Vegfaxxxb* transcripts as PCR artefacts: Extending findings to human

Interestingly, the sequence of the error-prone downstrem PCR primer (exemplified by O63I) identified through our empirical approach is virtuallly identical to the one proposed by Bates *et al*. [19] for specific amplification of *Vegfaxxxb* transcripts in human (O81I; Fig 7B). Indeed, *Vegfa* sequence is broadly conserved amongst vertebrates and Exon 8 and Exon 8b in *hVegfa* would both start with an AT motif (Fig 7B). We therefore tested the hypothesis that amplification of “*Vegfaxxxb*-like” PCR products may be a PCR artefact using human cDNA from HEK293 cells. As seen in Fig 7C, only faint PCR amplification was observed with the “exon 8b-specific” O81I primer, which would be entirely specific (20 bases / 20 bases) to “*Vegfaxxxb*-like” transcripts, including the 9 bases complementary to the 5’end of putative exon 8b (Fig 7C, sequence provided above the gel). In contrast, very robust amplification was obtained with O63I and O80I, which both carry a single mismatch to putative “*Vegfaxxxb*-like” transcripts. Interestingly, such point mutations result in primers that respectively possess 4 and 3 bases complementary to the proximal exon 8 motif (indicated in bold green in Fig 7B, Fig 7C), while O81I retains only 2 such bases (AT). Furthermore, the non-specific O90I primer (with a 5’-CACTCGGAT motif), which solely retains the AT motif consistently gave a stronger PCR amplification than the “exon 8b-specific” O81I primer.

In conclusion, our data are fully consistent with the notion that the weak PCR amplification obtained with the “*Vegfaxxxb*-specific” primer O81I arises from hybridization to the 5’ of exon 8 of angiogenic *Vegfaxxx* transcripts, partly due to the common AT motif. The stronger PCR amplification observed with O80I and O63I, which both bear mismatches to *Vegfaxxxb* sequence, yet support robust PCR amplification is in line with this hypothesis (i.e. amplification of *Vegfaxxx* transcripts), as is the consistent amplification obtained with O90I.

## Discussion

Our analysis of *Vegfa* expression in the PT/MBH of sheep, across seasons and reproductive states, using RT-PCR and analysis of RNAseq datasets, led to the identification of five *Vegfa* splice variants. These different splice variants have been previously identified in mouse and human tissues, which suggests phylogenetical conservation of *Vegfa* splicing events. All the sequences identified in this work correspond to *Vegfaxxx* transcripts, with no evidence for usage of an alternative splice site in exon8. This suggested that identification of anti-angiogenic *Vegfaxxxb* transcripts in prior studies may have been caused by methodological issues with PCR. We therefore devised an empirical approach, which led to the identification of the minimal sequence requirements of an error-prone “exon 8b-specific primer”.

The VEGFA165b isoform was initially identified by Bates *et al*. [14] in human renal carcinomas. In striking contrast to other VEGFA isoforms, which promote angiogenesis, functional tests showed that VEGFA165b inhibits angiogenesis. Since then, additional VEGFAxxxb isoforms have been identified, which are all splice variants that comprise or lack exons 6 and/or 7 [19]. In human, isoforms that include exon 8a harbor a CDKPRR sequence at their C-term while isoforms that include exon 8b bear the SLTRKD sequence. It has been proposed that this difference confers either angiogenic or anti-angiogenic properties (VEGFAxxx and VEGFAxxxb isoforms, respectively). Consequently, it has been suggested that the balance between these two families of VEGFA sets the angiogenic outcome [20,21]. This has considerable therapeutic implications as current anti-VEGFA treatments against multiple froms of cancer (Bevacizumab, Avastin®) or diabetic retinopathies and macular degeneration (Ranibizumab, Lucentis®) potentially target both pro- and anti-angiogenic forms of VEGFA [13,22]. Using an anti-VEGFAxxxb antibody developed against human isoforms and RT-PCR, Castle-Miller *et al*. [11] recently showed that anti-angiogenic VEGFAxxxb isoforms are also present within the ovine PT and PD. They reported that *Vegfa* transcripts undergo a seasonal switch in splicing, such that production of VEGFAxxxb is increased during the breeding season and leads to reduced angiogenesis. According to Castle-Miller *et al*., these anti-angiogenic *Vegfaxxxb* transcripts represent a substantial part of the total pool of *Vegfa* mRNA, as they are up to 4 times more abundant than *Vegfaxxx* mRNA in the ovine PT/ME [11]. This finding in sheep is in line with prior reports in various normal human tissues, wherein *Vegfaxxxb* transcripts may account for >70% of total *Vegfa* [20,21,23–25].

To investigate this splicing mechanism we took advantage of RNA samples and brain sections, which were previously used to establish the ovine MBH transcriptome across seasons and reproductive states (Fig 1, [10]). Our qPCR data in the MBH, using non-isoform specific PCR primers which allow amplification of *Vegfaxxx* and *Vegfaxxxb* isoforms, show that both *Vegfa* and *VegfR2* transcripts undergo a ~2-fold decrease from the non-breeding season (May) to the breeding season (August/November). This is suggestive of enhanced VEGFA signaling during long days. However, neither *Vegfa* nor *VegfR2* are acutely regulated by long days or by THX, which blocks the transition to anestrus at the end of winter. Consequently, there is no obvious correlation between levels of *Vegfa* or *VegfR2* and the reproductive state. Our ISH data refined the pattern of *Vegfa* expression, showing it is mostly expressed in the PT, and its expression is not affected by photoperiod or THX. The apparent inconsistency between qPCR and ISH data might be due to the relatively small transcriptional changes (~2-fold) and to the semi-quantitative nature and lower sensitivity of ISH compared to qPCR. Overall, our data are consistent with prior findings, by both Jabbour *et al*. [26] and Castle-Miller *et al*. [11] which show that total *Vegfa* mRNA and VEGFA protein levels do not display large seasonal variation in the PT.

We then used non-isoform-specific primers to allow RT-PCR amplification and characterization of both *Vegfaxxx* and putative *Vegfaxxxb* transcripts (Fig 3). The sequences identified are consistent with predicted sequences in databases and findings in other species, including human and mouse, which suggest phylogenetical conservation of *Vegfa* splicing events [13,16]. As assessed by RT-PCR and agarose gel electrophoresis, the transcripts with exon 6 skipping represent the major isoform (b2 band). We also identify four minor isoforms. In the shortest (b3 band), both exons 6 and 7 are skipped. For the three longer isoforms, exon 7 is retained while exon 6 may be retained, or undergo usage of cryptic donor or acceptor sites. This latter isoform (denoted b1c), which was not predicted in sheep, has recently been identified in human tissues [16]. Therefore, our approach successfully identified known, novel, and, rare *Vegfa* splice variants, all of which correspond to angiogenic *Vegfaxxx* isoforms as none of the 84 clones included exon 8b. These findings are not congruent with findings by Castle-Miller *et al*. that *Vegfaxxxb* transcripts are very abundant in the MBH [11].

In contrast, our data are fully consistent with findings by Eswarappa *et al*. [27], who used a similar RT-PCR approach in the cow, for which the proximal part of exon 8 is virtually identical to that of sheep. Echoing our findings, out of 74 cDNA clones sequenced, none included the putative exon 8b [27]. These authors proposed that anti-angiogenic isoforms may nevertheless arise as a consequence of programmed translational read-through leading to another isoform, dubbed VEGFA-x [27]. These isoforms may help explain the immunostaining obtained with the anti-VEGFAxxxb antibody, in spite of the absence or scarcity of specific *Vegfaxxxb* transcripts. However, the existence of this VEGFA-x isoform is disputed and its anti-angiogenic properties could not be replicated [28]. Furthermore, VEGFAxxxb isoforms have been found to be weakly angiogenic compared to VEGFAxxx isoforms, rather than anti-angiogenic [29,30]. Therefore, the existence of anti-angiogenic VEGFA isoforms remains controversial.

Interestingly, the anti-VEGFAxxxb antibody used by Castle-Miller et al [11] in sheep was initially developed against an epitope of 6 amino acids, SLTRKD, which corresponds to the C-Term shared by all putative human VEGFAxxxb isoforms. The C-Term sequence of presumptive VEGFAxxxb isoforms in sheep is slightly divergent, as these isoforms would harbor a CLTRKD sequence. Therefore, we infer that the epitope recognized by the anti-VEGFAxxxb antibody might be restricted to the sequence LTRKD. Considering that *Vegfaxxxb* transcripts and isoforms are non-existent or scarce, an alternative explanation for the VEGFAxxxb-like immunostaining might be cross-reactivity of the antibody with a protein bearing a similar epitope. To substantiate this hypothesis, we searched human protein databases using blastp (protein-protein Blast, see [31]), which led to the unambiguous identification of 10 proteins harboring an LTRKD motif (S1 Table). Interestingly, this anti-VEGFAxxxb antibody was also used to detect VEGFAxxxb isoforms in tissues from mouse [32] and rat [33,34], while these isoforms would harbor a distinct PLTGKTD motif at their C-term, as already pointed by others [15,35]. Taking these considerations into account, it is not possible to rule out that the anti-VEGFAxxxb antibody, which has been extensively used in multiple species to define the roles of VEGFAxxxb isoforms, may cross-react with a substantial number of proteins (>10). This calls for the development of more specific tools.

It has been proposed that the RT-PCR approach described above, making use of non-isoform-specific primers, may be biased towards amplification of certain *Vegfa* transcripts with respect to their abundance or potential issues with secondary structures [20]. We therefore designed isoform-specific primers, to specifically target and amplify *Vegfaxxxb* transcripts (Fig 4). We tested these primers on cDNA samples obtained across seasons and reproductive states. No PCR product could be obtained. Taken together, these data reinforce our conclusion that *Vegfaxxxb* transcripts are absent from the ewe MBH, or expressed to very low levels, i.e. below the sensitivity threshold of our RT-PCR methodology. As alluded to above, our findings in sheep echo multiple failed attempts by different research teams to reveal *Vegfaxxxb* mRNA in various animal models (cow, mouse and human) and using multiple methodologies [15,16,27,35–37]. In the most recent study, Bridgett *et al*. [16] used publicly available RNA-seq datasets to search for *Vegfaxxxb* transcripts in human tissues. Their extensive analysis identified >40000 *Vegfa* transcripts that cover the junction between exon 7 and exon 8 in >10 different human tissues. None of the reads supported the existence of an exon 8b splice site, hence the existence of *Vegfaxxxb* mRNA [16]. Our current analysis of independent MBH/PT RNA-seq datasets, which were generated in ewes [10] and rams [9], fully concurs with findings by Bridgett *et al*. in human tissues (Table 2). This provides further support to the notion that previous detection of *Vegfaxxxb* mRNA may be accounted for by methodological issues. Indeed, Harris et al [15] had already dedicated a thorough study to the identification of *Vegfaxxxb* in mouse and human tissues and cells and concluded that detection of *Vegfaxxxb* mRNA might arise as a PCR artefact through “5’ tailing”.

In reply to the study by Harris *et al*. [15], Bates *et al*. [19] argued that inability to detect *Vegfaxxxb* mRNA was due to inadequate primer design and lack of appropriate controls. According to Bates *et al*., the 3’end of the adequate *Vegfaxxxb*-specific primer includes 11 bases complementary to exon 7, while the 9 bases at the 5’end are complementary to the putative exon 8b [19]. The authors validated their primer specificity using a construct that bears the putative *Vegfaxxxb* sequence. However, the authors did not valide their primers on tissue cDNA, nor did they assess whether the 9 bases on the 5’end provide specificity.

With these considerations in mind, we used a step-by-step approach to identify an “exon 8b-specific” downstream primer able to yield a “*Vegfaxxxb*-like” PCR product (Figs 5 and 6). Collectively, our experiments define the minimal requirements for such a primer: a 3’end with 11 bases complementary to exon 7 followed by the first 2–3 bases of exon 8, which together provide specificity, then a stretch of random nucleotides, which increases the melting temperature of the oligonucleotide (Fig 7). This error-prone primer appears identical to the one proposed by Bates *et al*. [19] to specifically detect *Vegfaxxxb* RNA in human tissues, which questions interpretation of prior findings. Overall, the RT-PCR approaches we developed using sheep MBH are quite similar to those developed for mouse and human tissues and cells (including HEK293) by Harris *et al*. [15] and our conclusions concur with theirs: “VEGFxxxb products were consistently amplified only when the reverse primer contained more bases of complementary sequence to exon 7 or exon 5 as opposed to exon 8b (i.e. 5’ tailing).” In this study [15], amplification of *Vegfaxxxb*-like transcripts in mouse and human could be obtained when the number of bases complementary to exon 7 or exon 5 was equal or >13, which is in line with our current findings in sheep.

## Conclusions

Our results (i) show that the ovine PT may use enhanced VEGFA signaling during long days, the consequences of which in terms of breeding control–if any–remain to be established, (ii) reveal the existence of at least five *Vegfa* splice variants in the ovine MBH, all of which belong to the pro-angiogenic *Vegfaxxx* family and (iii) unveil issues in *Vegfaxxxb* detection methodology by RT-PCR. In conclusion, our findings do not support the existence of anti-angiogenic *Vegfaxxxb* isoforms in the ovine PT/MBH and shed new light on the interpretation of prior studies, which claimed to identify *Vegfaxxxb* isoforms by RT-PCR.

## Supporting information

S1 Fig Dissection procedure. A/ Procedure for dissecting the MBH block. The two pictures on the left are ventral views of the ovine brain; the two pictures on the right are coronal slices. Details are provided in the panel. B/ Procedure for dissecting the PD block.
(PDF)

S2 Fig Visualization of the ovine *Vegfa* gene locus with known transcripts and a summary of all identified splice junctions (see Table 2 for details). The *Ovis aries* v4.0 genome and annotation was uploaded into the Broad Institute IGV browser (http://software.broadinstitute.org/software/igv/). STAR junction tables were converted to bed format using a custom bash script and the resulting bed file was uploaded along with the BAM and BAM index files for a representative sample.
(PDF)

S1 Table Use of blastp reveals a list of 10 human proteins with a CLTKD motif. (PDF)

S1 Document NC3Rs ARRIVE guidelines checklist. (PDF)

## References

[1] Hanon EA, Lincoln GA, Fustin JM, Dardente H, Masson-Pevet M, Morgan PJ, et al Ancestral TSH mechanism signals summer in a photoperiodic mammal. Curr Biol. 2008;18: 1147–1152. doi: 10.1016/j.cub.2008.06.076 18674911 10.1016/j.cub.2008.06.076

[2] Dardente H, Wyse CA, Birnie MJ, Dupre SM, Loudon AS, Lincoln GA, et al A molecular switch for photoperiod responsiveness in mammals. Curr Biol. 2010;20: 2193–2198. doi: 10.1016/j.cub.2010.10.048 21129971 10.1016/j.cub.2010.10.048

[3] Dardente H, Hazlerigg DG, Ebling FJ. Thyroid hormone and seasonal rhythmicity. Front Endocrinol (Lausanne). 2014;5: 19. 24616714 10.3389/fendo.2014.00019 PMC3935485

[4] Ikegami K, Liao XH, Hoshino Y, Ono H, Ota W, Ito Y, et al Tissue-specific posttranslational modification allows functional targeting of thyrotropin. Cell Rep. 2014;9: 801–810. doi: 10.1016/j.celrep.2014.10.006 25437536 10.1016/j.celrep.2014.10.006 PMC4251493

[5] Yoshimura T, Yasuo S, Watanabe M, Iigo M, Yamamura T, Hirunagi K, et al Light-induced hormone conversion of T4 to T3 regulates photoperiodic response of gonads in birds. Nature. 2003;426: 178–181. doi: 10.1038/nature02117 14614506 10.1038/nature02117

[6] Barrett P, Ebling FJ, Schuhler S, Wilson D, Ross AW, Warner A, et al Hypothalamic thyroid hormone catabolism acts as a gatekeeper for the seasonal control of body weight and reproduction. Endocrinology. 2007;148: 3608–3617. doi: 10.1210/en.2007-0316 17478556 10.1210/en.2007-0316

[7] Nakao N, Ono H, Yamamura T, Anraku T, Takagi T, Higashi K, et al Thyrotrophin in the pars tuberalis triggers photoperiodic response. Nature. 2008;452: 317–322. doi: 10.1038/nature06738 18354476 10.1038/nature06738

[8] Dardente H. Circannual Biology: The Double Life of the Seasonal Thyrotroph. Curr Biol. 2015;25: R988–91. doi: 10.1016/j.cub.2015.09.002 26485373 10.1016/j.cub.2015.09.002

[9] Wood SH, Christian HC, Miedzinska K, Saer BR, Johnson M, Paton B, et al Binary Switching of Calendar Cells in the Pituitary Defines the Phase of the Circannual Cycle in Mammals. Curr Biol. 2015;25: 2651–2662. doi: 10.1016/j.cub.2015.09.014 26412130 10.1016/j.cub.2015.09.014 PMC4612467

[10] Lomet D, Cognie J, Chesneau D, Dubois E, Hazlerigg D, Dardente H. The impact of thyroid hormone in seasonal breeding has a restricted transcriptional signature. Cell Mol Life Sci. 2018;75: 905–919. doi: 10.1007/s00018-017-2667-x 28975373 10.1007/s00018-017-2667-x PMC11105383

[11] Castle-Miller J, Bates DO, Tortonese DJ. Mechanisms regulating angiogenesis underlie seasonal control of pituitary function. Proc Natl Acad Sci U S A. 2017;114: E2514–E2523. doi: 10.1073/pnas.1618917114 28270617 10.1073/pnas.1618917114 PMC5373413

[12] Ferrara N, Gerber HP, LeCouter J. The biology of VEGF and its receptors. Nat Med. 2003;9: 669–676. doi: 10.1038/nm0603-669 12778165 10.1038/nm0603-669

[13] Ferrara N. Vascular endothelial growth factor: basic science and clinical progress. Endocr Rev. 2004;25: 581–611. doi: 10.1210/er.2003-0027 15294883 10.1210/er.2003-0027

[14] Bates DO, Cui TG, Doughty JM, Winkler M, Sugiono M, Shields JD, et al VEGF165b, an inhibitory splice variant of vascular endothelial growth factor, is down-regulated in renal cell carcinoma. Cancer Res. 2002;62: 4123–4131. 12124351

[15] Harris S, Craze M, Newton J, Fisher M, Shima DT, Tozer GM, et al Do anti-angiogenic VEGF (VEGFxxxb) isoforms exist? A cautionary tale. PLoS One. 2012;7: e35231 doi: 10.1371/journal.pone.0035231 22567098 10.1371/journal.pone.0035231 PMC3342274

[16] Bridgett S, Dellett M, Simpson DA. RNA-Sequencing data supports the existence of novel VEGFA splicing events but not of VEGFAxxxb isoforms. Sci Rep. 2017;7: 58-017-00100-3. 10.1038/s41598-017-00100-3 PMC5427905 28246395

[17] Karsch FJ, Bittman EL, Foster DL, Goodman RL, Legan SJ, Robinson JE. Neuroendocrine basis of seasonal reproduction. Recent Prog Horm Res. 1984;40: 185–232. 6385166 10.1016/b978-0-12-571140-1.50010-4

[18] Dobin A, Davis CA, Schlesinger F, Drenkow J, Zaleski C, Jha S, Batut P, Chaisson M, Gingeras TR. STAR: ultrafast universal RNA-seq aligner. Bioinformatics. 2013; 29:15–21 doi: 10.1093/bioinformatics/bts635 23104886 10.1093/bioinformatics/bts635 PMC3530905

[19] Bates DO, Mavrou A, Qiu Y, Carter JG, Hamdollah-Zadeh M, Barratt S, et al Detection of VEGF-A(xxx)b isoforms in human tissues. PLoS One. 2013;8: e68399 doi: 10.1371/journal.pone.0068399 23935865 10.1371/journal.pone.0068399 PMC3729684

[20] Qiu Y, Hoareau-Aveilla C, Oltean S, Harper SJ, Bates DO. The anti-angiogenic isoforms of VEGF in health and disease. Biochem Soc Trans. 2009;37: 1207–1213. doi: 10.1042/BST0371207 19909248 10.1042/BST0371207 PMC2882696

[21] Nowak DG, Woolard J, Amin EM, Konopatskaya O, Saleem MA, Churchill AJ, et al Expression of pro- and anti-angiogenic isoforms of VEGF is differentially regulated by splicing and growth factors. J Cell Sci. 2008;121: 3487–3495. doi: 10.1242/jcs.016410 18843117 10.1242/jcs.016410 PMC2613349

[22] Wells JA, Glassman AR, Ayala AR, Jampol LM, Aiello LP, et al Aflibercept, bevacizumab, or ranibizumab for diabetic macular edema. N Engl J Med. 2015;372: 1193–1203. doi: 10.1056/NEJMoa1414264 25692915 10.1056/NEJMoa1414264 PMC4422053

[23] Perrin RM, Konopatskaya O, Qiu Y, Harper S, Bates DO, Churchill AJ. Diabetic retinopathy is associated with a switch in splicing from anti- to pro-angiogenic isoforms of vascular endothelial growth factor. Diabetologia. 2005;48: 2422–2427. doi: 10.1007/s00125-005-1951-8 16193288 10.1007/s00125-005-1951-8

[24] Varey AH, Rennel ES, Qiu Y, Bevan HS, Perrin RM, Raffy S, et al VEGF 165 b, an antiangiogenic VEGF-A isoform, binds and inhibits bevacizumab treatment in experimental colorectal carcinoma: balance of pro- and antiangiogenic VEGF-A isoforms has implications for therapy. Br J Cancer. 2008;98: 1366–1379. doi: 10.1038/sj.bjc.6604308 18349829 10.1038/sj.bjc.6604308 PMC2361696

[25] Bevan HS, van den Akker NM, Qiu Y, Polman JA, Foster RR, Yem J, et al The alternatively spliced anti-angiogenic family of VEGF isoforms VEGFxxxb in human kidney development. Nephron Physiol. 2008;110: p57–67. doi: 10.1159/000177614 19039247 10.1159/000177614 PMC2635558

[26] Jabbour HN, Boddy SC, Lincoln GA. Pattern and localisation of expression of vascular endothelial growth factor and its receptor flt-1 in the ovine pituitary gland: expression is independent of hypothalamic control. Mol Cell Endocrinol. 1997;134: 91–100. 9426152 10.1016/s0303-7207(97)00158-5

[27] Eswarappa SM, Potdar AA, Koch WJ, Fan Y, Vasu K, Lindner D, et al Programmed translational readthrough generates antiangiogenic VEGF-Ax. Cell. 2014;157: 1605–1618. doi: 10.1016/j.cell.2014.04.033 24949972 10.1016/j.cell.2014.04.033 PMC4113015

[28] Xin H, Zhong C, Nudleman E, Ferrara N. Evidence for Pro-angiogenic Functions of VEGF-Ax. Cell. 2016;167: 275–284.e6. doi: 10.1016/j.cell.2016.08.054 27662093 10.1016/j.cell.2016.08.054

[29] Catena R, Larzabal L, Larrayoz M, Molina E, Hermida J, Agorreta J, et al VEGF(1)(2)(1)b and VEGF(1)(6)(5)b are weakly angiogenic isoforms of VEGF-A. Mol Cancer. 2010;9: 320-4598-9-320. 10.1186/1476-4598-9-320 PMC3022671 21194429

[30] Guyot M, Hilmi C, Ambrosetti D, Merlano M, Lo Nigro C, Durivault J, et al Targeting the pro-angiogenic forms of VEGF or inhibiting their expression as anti-cancer strategies. Oncotarget. 2017;8: 9174–9188. doi: 10.18632/oncotarget.13942 27999187 10.18632/oncotarget.13942 PMC5354723

[31] Altschul SF, Gish W, Miller W, Myers EW, Lipman DJ. Basic local alignment search tool. J Mol Biol. 1990;215: 403–410. doi: 10.1016/S0022-2836(05)80360-2 2231712 10.1016/S0022-2836(05)80360-2

[32] Zhao M, Shi X, Liang J, Miao Y, Xie W, Zhang Y, et al Expression of pro- and anti-angiogenic isoforms of VEGF in the mouse model of oxygen-induced retinopathy. Exp Eye Res. 2011;93: 921–926. doi: 10.1016/j.exer.2011.10.013 22067127 10.1016/j.exer.2011.10.013

[33] Ergorul C, Ray A, Huang W, Darland D, Luo ZK, Grosskreutz CL. Levels of vascular endothelial growth factor-A165b (VEGF-A165b) are elevated in experimental glaucoma. Mol Vis. 2008;14: 1517–1524. 18728749 PMC2518529

[34] Artac RA, McFee RM, Smith RA, Baltes-Breitwisch MM, Clopton DT, Cupp AS. Neutralization of vascular endothelial growth factor antiangiogenic isoforms is more effective than treatment with proangiogenic isoforms in stimulating vascular development and follicle progression in the perinatal rat ovary. Biol Reprod. 2009;81: 978–988. doi: 10.1095/biolreprod.109.078097 19605786 10.1095/biolreprod.109.078097 PMC2770023

[35] Guyot M, Pagès G. VEGF splicing and the role of VEGF splice variants: from physiological-pathological conditions to specific pre-mRNA splicing VEGF signaling: Methods and Protocols, Methods in Molecular Biology, vol 1332 Springer Science Business Media New York 2015: 3–23. 10.1007/978-1-4939-2917-7_1 26285742

[36] Gustafsson T, Ameln H, Fischer H, Sundberg CJ, Timmons JA, Jansson E. VEGF-A splice variants and related receptor expression in human skeletal muscle following submaximal exercise. J Appl Physiol (1985). 2005;98: 2137–2146. doi: 10.1152/japplphysiol.01402.2004 15661835 10.1152/japplphysiol.01402.2004

[37] Dokun AO, Annex BH. The VEGF165b "ICE-o-form" puts a chill on the VEGF story. Circ Res. 2011;109: 246–247. doi: 10.1161/CIRCRESAHA.111.249953 21778432 10.1161/CIRCRESAHA.111.249953 PMC3196354

