# Supplementary material for: Anti-angiogenic VEGFAxxxb transcripts are not expressed in the medio-basal hypothalamus of the seasonal sheep
Source: PLoS One. 2018 May 10;13(5):e0197123. doi: 10.1371/journal.pone.0197123 (PMC5944957; doi:10.1371/journal.pone.0197123)
Supplement: S1 Fig — A/ Procedure for dissecting the MBH block. The two pictures on the left are ventral views of the ovine brain; the two pictures on the right are coronal slices. Details are provided in the panel. B/ Procedure for dissecting the PD block. (PDF) [file pone.0197123.s001.pdf]

A

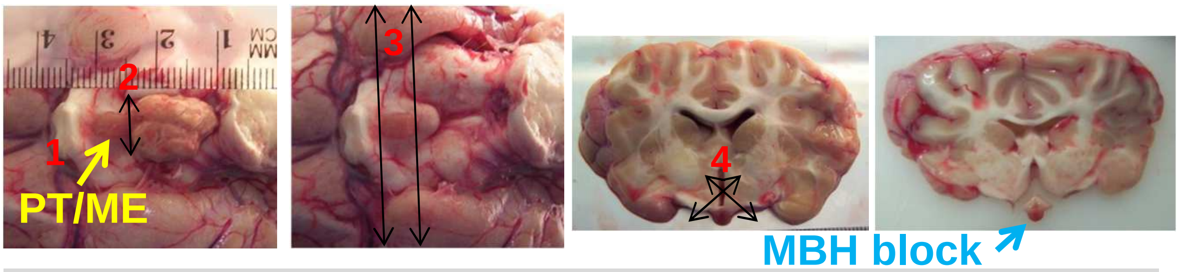

1/ Length of PT/ME = 5-6mm, remove surrounding conjonctive tissue.
2/ Cut the PD out at the PT/PD junction (*zona tuberalis, zt*).
3/ Make a slice covering the rostro-caudal extent of the PT/ME, following arrows.
4/ Prepare the MBH block: from the bottom of the 3V: 3-4mm lateral and 4-5mm dorsal, cut following arrows.

B

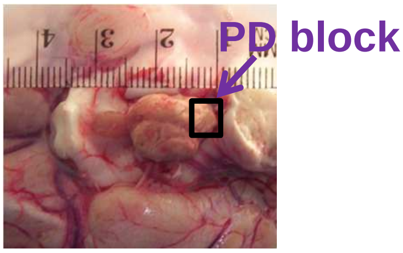

Supplemental Figure S1
