# Supplementary material for: Anti-angiogenic VEGFAxxxb transcripts are not expressed in the medio-basal hypothalamus of the seasonal sheep
Source: PLoS One. 2018 May 10;13(5):e0197123. doi: 10.1371/journal.pone.0197123 (PMC5944957; doi:10.1371/journal.pone.0197123)
Supplement: S1 Table — (PDF) [file pone.0197123.s003.pdf]

GenBank Accession N°	Protein name	LTRKD motif location
NM_001109662	HECT domain E3 ubiquitin protein ligase 4 (HECTD4)	3233-3237
NM_001897	Chondroitin sulfate proteoglycan 4 (CSPG4)	1058-1062
NM_006071	Polycystin family receptor for egg jelly (PKDREJ)	1352-1356
NM_014272	ADAM metallopeptidase with thrombospondin type 1 motif 7 (ADAMTS7)	341-345
EAX08997	Dedicator of cytokinesis 9, isoform CRA_d	1649-1653
XM_011519428	PREDICTED: Disco interacting protein 2 homolog C (DIP2C)	477-481
NM_015072	Tubulin tyrosine ligase like 5 (TTLL5)	128-132
XM_017011995	Amphiphysin (AMPH), transcript variant X1	706-710
NM_172364	Calcium voltage-gated channel auxiliary subunit alpha2delta 4 (CACNA2D4)	709-713
XM_011514460	Zinc finger protein 451 (ZNF451), transcript variant X1	512-516

Supplemental Table1
