# Supplementary material for: Anti-angiogenic VEGFAxxxb transcripts are not expressed in the medio-basal hypothalamus of the seasonal sheep
Source: PLoS One. 2018 May 10;13(5):e0197123. doi: 10.1371/journal.pone.0197123 (PMC5944957; doi:10.1371/journal.pone.0197123)
Supplement: S2 Fig — The Ovis aries v4.0 genome and annotation was uploaded into the Broad Institute IGV browser (http://software.broadinstitute.org/software/igv/). STAR junction tables were converted to bed format using a custom bash script and the resulting bed file was uploaded along with the BAM and BAM index files for a representative sample. (PDF) [file pone.0197123.s002.pdf]

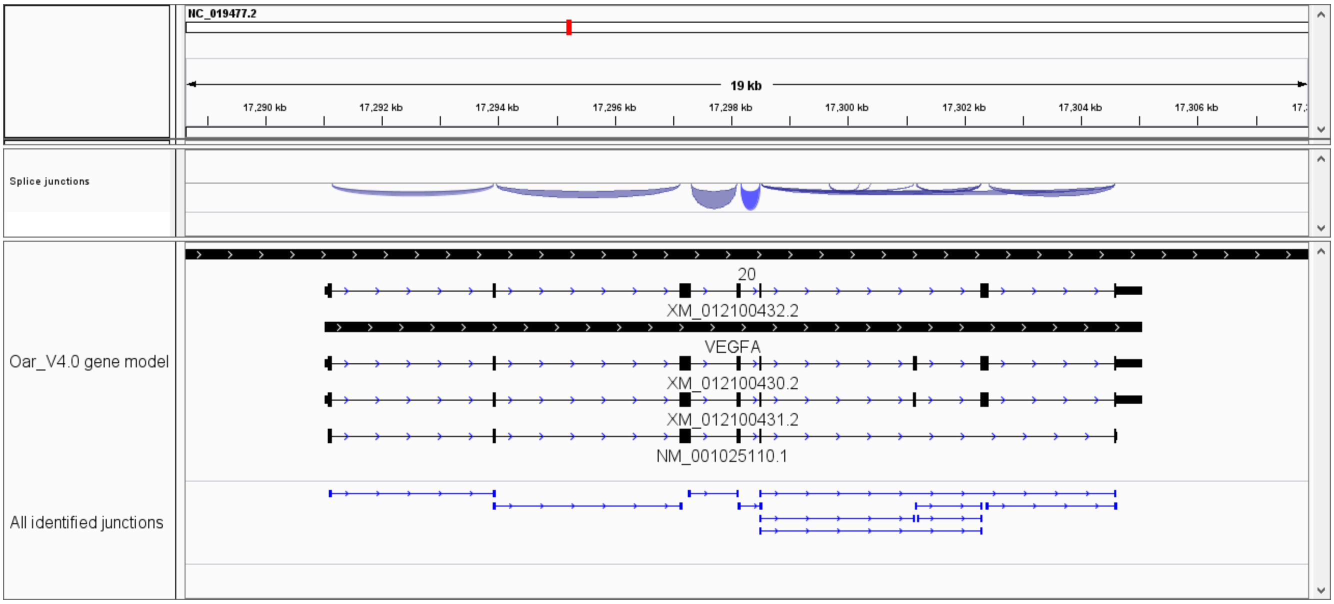

Supplemental Figure S2
